# Preoperative planning and surgical technique for optimizing internal fixation of posterior malleolar fractures: CT versus standard radiographs

**DOI:** 10.1186/s13018-020-01637-2

**Published:** 2020-03-26

**Authors:** Ezequiel Palmanovich, Nissim Ohana, Eyal Yaacobi, David Segal, Hetsroni Iftach, Zachary T. Sharfman, Matias Vidra, Ran Atzmon

**Affiliations:** 1 Orthopedic Department, Meir Hospital, Sapir Medical Center affiliated with the Sackler Faculty of Medicine, Tel Aviv University, 56 Tchernichovsky St, Kfar Saba, Israel; 2 Department of Orthopaedic Surgery, Montefiore Medical Center, Albert Einstein College of Medicine, 1300 Morris Park Ave, The Bronx, NY 10461 USA; 3 Department of Orthopaedic Surgery, Tel Aviv Medical Center affiliated with the Sackler Faculty of Medicine, Tel Aviv University, Weizmann St. 6, Tel Aviv-Yafo, Israel; 4 Department of Orthopaedic Surgery, Assuta Medical Center affiliated with the Faculty of Health and Science, Ben Gurion University of the Negev, Menachem Begin Blvd. 126, Ashdod, Israel

**Keywords:** Preoperative planning, Surgical technique, Posterior malleolar fracture, Computed tomography, X-ray

## Abstract

**Background:**

A proper reduction and internal fixation of posterior malleolar fractures can be challenging, as intraoperative fluoroscopy often underestimates the extent of the fracture. Our aim was to assess the value of a modified classification system for posterior malleolar fractures, which is based on computed tomography (CT) images, optimizing screw trajectory during fluoroscopic-guided surgery, and to compare it to the Lauge-Hansen classification system to the CT-based classification.

**Methods:**

A retrospective review of all ankle fracture operations from January 2014 to December 2016 was performed. Fractures were included if a CT scan was performed within 1 week of the surgery, and the posterior malleolar fragment occupied one third or more of the antero-posterior talar surface or jeopardize the ankle stability. Eighty-five adult ankle fractures with posterior malleolar fragments were included in this study. Fractures were categorized into one of three types, namely “postero-lateral,” “postero-medial,” or “postero-central,” according to the location of the fracture fragment on axial CT image. An optimal trajectory angle for a single-lag screw fixation was measured on the CT cut between a central antero-posterior line and the line intersecting the posterior fragment perpendicular to the major fracture line. Mean trajectory angles were calculated for each fracture type. Fractures were also categorized according to the Lauge-Hansen system.

**Results:**

The mean trajectory angle was 21° lateral for “postero-lateral” fragments, 7° lateral for “postero-central” fragments, and 28° medial for “postero-medial” fragments (*p* < 0.01 for comparisons among the groups). The range of trajectory angles within each group was about 10°, as compared to about 20° within each Lauge-Hansen type. There were no differences in trajectory angle among the Lauge-Hansen groups (*p* > 0.05 for all comparisons).

**Conclusions:**

There are 3 distinct anatomic subgroups of posterior malleolar fragments, each with an ideal screw trajectory that needs to be used in order to achieve an optimal reduction and fixation.

## Background

Ankle fractures are among the most common types of fractures treated by orthopedic surgeons [1]. The incidence of ankle fractures is reported to be 187 fractures per 100,000 people each year [2]. Sixty to 70% of these injuries are unimalleolar fractures, and 15 to 20% are bimalleolar fractures involving both the medial and lateral malleoli. Seven to 12% of all ankle fractures can be classified as trimalleolar fractures, which involve the posterior malleolus in addition to the medial and lateral malleoli [3, 4]. A posterior malleolar fracture constitutes 7 to 44% of all ankle fractures [3, 5–7]. While less than 1% of ankle fractures are isolated posterior malleolar fractures, the majority of this fracture commonly occurs in relation to lateral or medial malleolar fractures [1, 8]. Indications for surgery in cases of posterior malleolar fractures are subject to ongoing debate [9, 10]. Typically, the size of the posterior malleolar fragment is taken into account when considering operative fixation. Some investigators recommend surgical intervention when the posterior fragment is measured to be one third to one fourth of the antero-posterior curvature of the articular surface of the tibial plafond [1–6]. Other authors support surgical intervention only when the fragment involves more than 50% of the articular surface [11]. Another common indication for surgery is a posterior malleolar fracture in combination with lateral malleolar fractures [12]. In general, if posterior subluxation of the tibiotalar joint is seen in the presence of a posterior malleolus fracture operative fixation is indicated. Finally, when the articular surface of the distal tibia is involved, or the integrity of the syndesmosis is jeopardized, surgical intervention should be considered [9, 10, 13]. When reduction and internal fixation of a posterior malleolar fracture are indicated, achieving anatomic fixation can be challenging. While medial and lateral malleolar fractures can be reduced anatomically during surgery, intraoperative fluoroscopic anterior posterior (AP), lateral, and mortise views generally underestimate the extent of the posterior malleolar fractures [12, 14]. Computed tomography (CT) imaging significantly improved the accuracy of assessing the extent of posterior malleolar fractures [9] using Haraguchi et al.’s suggested classification [12, 14]. Although some surgeons may consider it unnecessary, intraoperative CT [15] can be advantageous in the assessment of posterior malleolar reduction over fluoroscopic guidance alone. However, intraoperative CT is not preferred, or available, and preoperative CT imaging can be used for preoperative planning in primary cases. Some surgeons may employ early postoperative CT to assess the accuracy of their reduction.

Since 2009, the authors of this paper routinely performed early postoperative CT assessment of ankle fractures that involved posterior malleolar fractures after close or open reduction and internal fixation, in order to evaluate the accuracy of the reduction [16]. Despite the use of intraoperative fluoroscopy in order to achieve an acceptable reduction on image intensifier, many early postoperative CTs demonstrated an inaccurate reduction of the posterior malleolus in the eyes of the lead surgeon. In approximately two thirds of the cases reviewed, posterior malleolar fractures were under-reduced due to misplaced screws and poor understanding of the fracture pattern. It was previously shown that even limited posterior malleolar fragments are indicative of poor prognosis and portend an increased risk for the development of ankle arthritis [6, 14, 17–19]. In light of these findings, the objective of this study was to characterize posterior malleolar fractures based on preoperative X-ray using the standard Lauge-Hansen classification system [20], to characterize these fractures based on the location of the posterior malleolar fragments on preoperative CT imaging, and (3) to assess the posterior malleolar fracture different patterns to accurately optimized screw trajectory. Additionally, this study aimed to appraise the adequacy between the Lauge-Hansen classification system for predicting the appropriate screw trajectory in ankle fractures with a posterior malleolar component for each fracture’s type.

## Materials and methods

We performed a retrospective comparative cohort study of prospectively gathered data from a single center. The study was approved by the local institutional review board and took place between January 2014 and December 2016. A single fellowship-trained orthopedic surgeon performed all radiographic measurements. Eighty-five patients with posterior malleolar fractures were included in this study. Inclusion criteria were (1) skeletal maturity; (2) the presence of a posterior malleolar fragment that occupied one third or more of the antero-posterior curvature of the articular surface of the tibial plafond, the presence of a posterior malleolar fracture associated with tibiotalar dislocation, and non-reduced syndesmosis or the posterior inferior tibiofibular ligament (PITFL) which is connected to the posterior malleolar fragment; (3) the presence of a non-comminuted fracture without intra-articular fragments that would prevent screw fixation; (4) preoperative X-ray; (5) all the CT scan examinations which were performed during hospitalization; and (6) pre- and a post-operative CT scans which were performed within 1 week after internal fixation of the posterior malleolar fractures, in a case operative treatment was indicated. Pilon fracture of the distal tibia was excluded from the study.

**Fig. 1:**
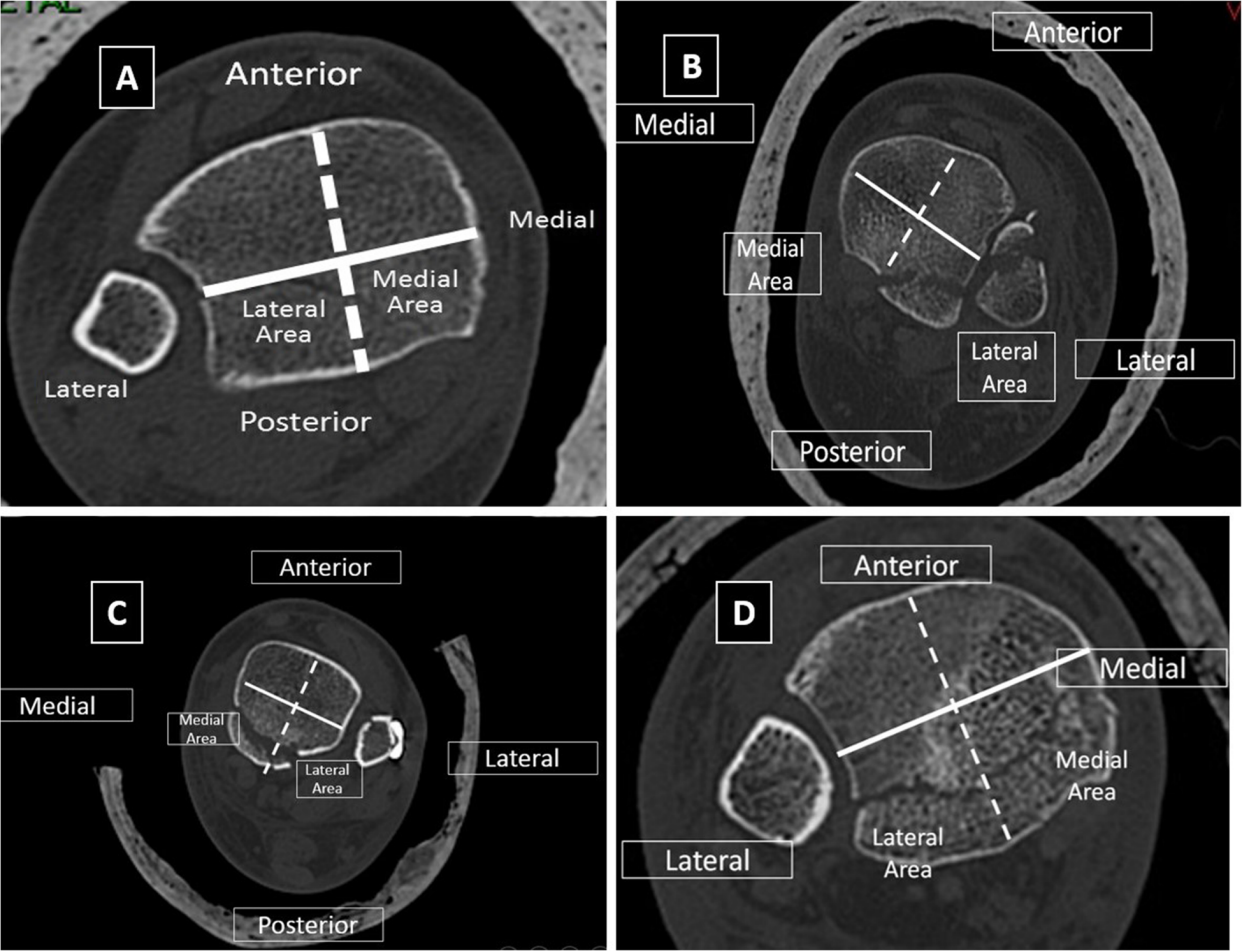
A method for CT-based classification of posterior malleolar fractures. **a** Superimposed on an axial CT image of the ankle, a solid line is subtended between the medial and lateral malleoli. Then, a second (dashed) line is drawn perpendicularly to the first intermalleolar line, dividing the posterior malleolus area into medial and lateral segments. **b**–**d** CT images showing the different fracture types: postero-lateral (**b**), postero-medial (**c**), and postero-central (**d**)

In order to categorize the posterior malleolus fracture, two methods were used. In the first, we further developed the previously describe classification by Haraguchi et al. [14] which divided the posterior malleolus into 3 main types based on CT scan: posterolateral-oblique, small-shell type, and medial-extension which involves the medial malleolus in 20% of the cases. In our modified classification, the axial CT cut that demonstrated the largest posterior fragment was selected. The horizontal line was then drawn between the most prominent part of the medial malleolus and the Chaput tubercle, dividing the tibia into anterior and posterior segments. The posterior segment was then divided by a central line, perpendicular to the bimalleolar line, into medial and lateral sub-segments (Fig. 1a), thus creating a 4-quadrant grid. Each posterior malleolar fracture was then categorized into one of the three groups based on the fragment’s location: posterior fragments occupying predominantly the posterior-lateral sub-segment were classified as “postero-lateral” (not equivalent to a Volkmann fragment) (Fig. 1b), fragments occupying predominantly the posterior-medial segment were categorized as “postero-medial” (Fig. 1c), and fragments occupying both medial and lateral sub-segments were categorized as “postero-central” (Fig. 1d). The shape of the fragments was also noted.

**Fig. 2:**
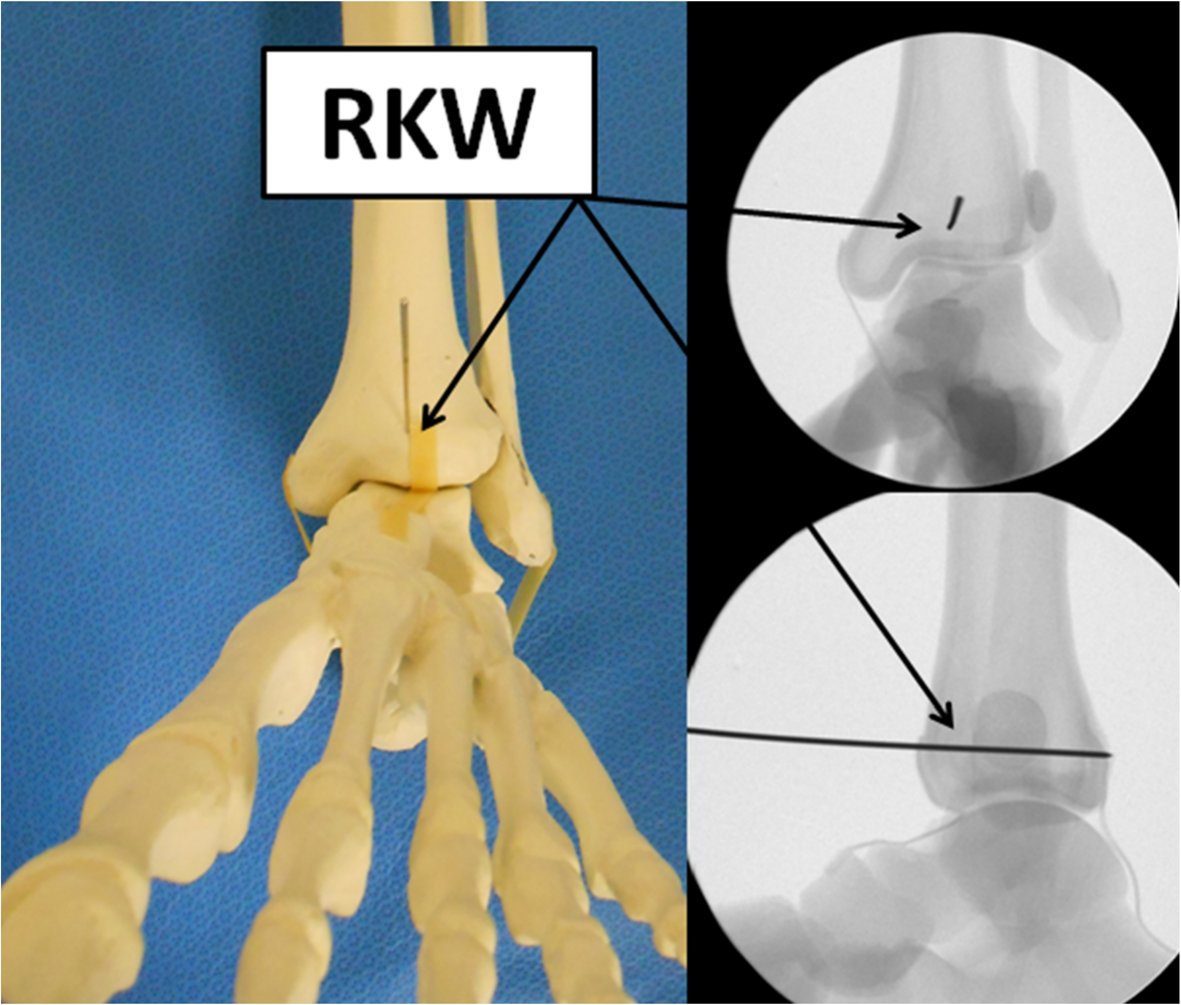
A reference K-wire (rKW) is inserted midway between the medial malleolus and the Chaput prominence, parallel to the articular surface in antero-posterior direction

Next, we used our superimposed images to plan and predict the optimal trajectory for posterior fragment fixation with a single posterior-to-anterior percutaneous screw. First, the most anterior prominent part of the tibial cortex located midway between the most prominent part of the medial malleolus and the Chaput tubercle on the lateral side of the tibia was the identified case on the CT cuts. These two points were chosen because, in practice, they are easily found by palpation and can therefore be used intraoperatively. Second, a reference line was marked perpendicular to the bimalleolar line. Third, a trajectory line, perpendicular to the fracture line, was marked from the most prominent anterior part toward the posterior part of the fragment on the axial CT images. The angle formed between this line and the line perpendicular to the bimalleolar line was defined as the “trajectory angle” (Fig. 2). This angle was measured and averaged for each fragment type.

The second classification method we used was according to the Lauge-Hansen classification system [20] and the Arimoto and Forrester ankle fracture algorithm [21]. All was done by one investigator, based on standard trauma series radiographs.

### Surgical technique

**Fig. 3:**
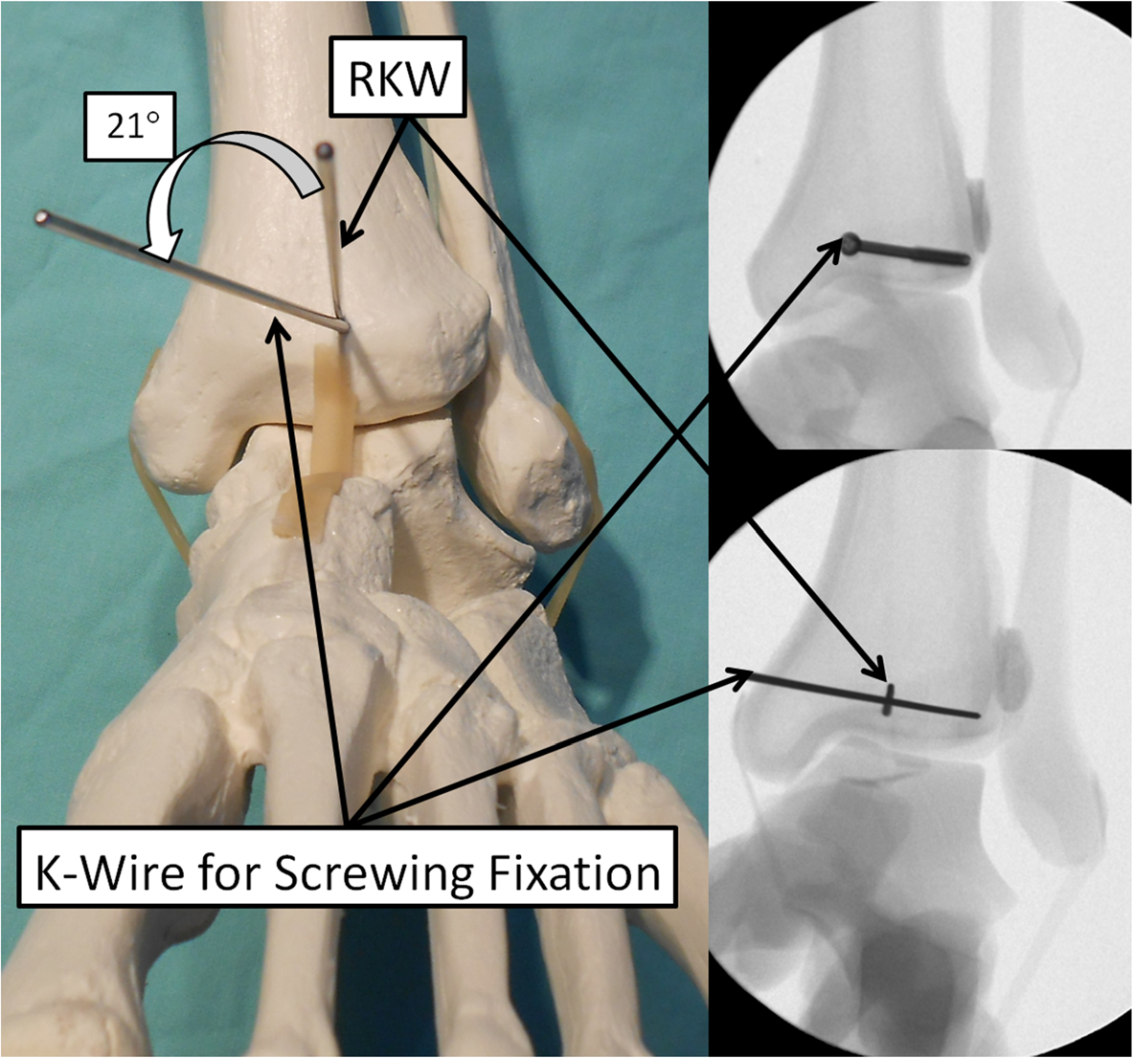
A second K-wire is inserted as a guide-pin for the screw in the correct trajectory angle in order to intersect and adequately stabilize the posterior malleolar fragment. The direction of this K-wire corresponds to the mean trajectory angle of the specific fracture type calculated on the axial CT cut

**Fig. 4:**
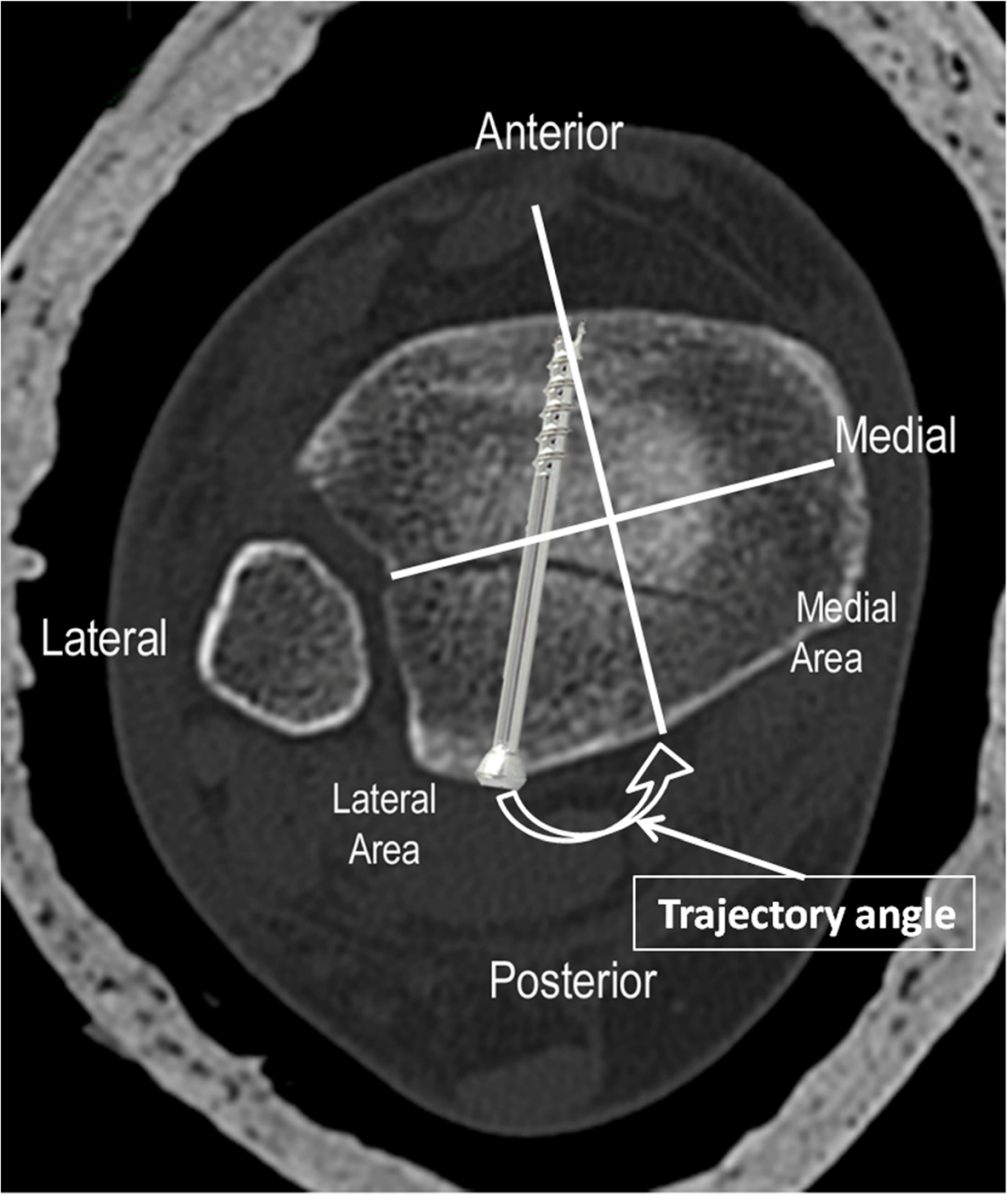
Axial CT image demonstrating the trajectory angle and ideal screw lag placement in postero-lateral type posterior malleolar fracture

In order to obtain optimized screw trajectory for internal fixation based on fragment classification (i.e., “postero-lateral,” “postero-medial,” or “postero-central”), a reference Kirschner wire (rKW) was first inserted from the most anteriorly prominent and palpable part on the tibial cortex, midway between the medial prominence of the medial malleolus and the Chaput tubercle, in an anterior to posterior direction under antero-posterior fluoroscopic imaging (Fig. 2). Using lateral fluoroscopic imaging, the rKW was then advanced posteriorly while maintained parallel to the ankle joint line. The trajectory Kirschner wire (tKW) was then inserted adjacent to the rKW, at an angle corresponding to the mean trajectory angle for the specific morphological type of the posterior malleolar fragment, parallel to the joint line (Fig. 3). A posterior-to-anterior-cannulated screw was then inserted over the tKW in order to stabilize the posterior fragment (Fig. 4). It is advisable to make a small skin incision followed by a minimal blunt dissection prior to the KW and screw insertion, based on the planned hardware route, in order to avoid damaging the surrounding neuro-vascular structures.

### Statistical analysis

Descriptive statistics are presented as mean values with ranges. Student *t* tests were used to examine the differences between the trajectory angles among each of the three CT-based fracture classification groups and for each of the Lauge-Hansen fracture types. An alpha error of 0.05 was considered statistically significant.

## Results

A total of 85 patients who underwent surgical internal fixation for a posterior malleolar fracture between January 2014 and December 2016 met all the inclusion criteria for this study. This included 62 females and 23 males with a mean age of 53.4 years (range, 17–89 years). In 60% of cases (51 ankles), the right ankle was involved. Sixty-one cases were classified as “postero-lateral,” four cases as “postero-medial,” and 20 cases as “postero-central,” with mean trajectory angles of 21° (17–26°), 28° (25–31°), and 7° (2–13°), respectively. The measurements are summarized in Table 1. Trajectory angles were significantly different between all the fracture types (*p* < 0.01). More than 60% of the cases were either supination-lateral rotation type IV, or pronation-abduction type III, when classified according to the Lauge-Hansen classification (Table 2). No statistically significant differences were found comparing the average trajectory angle among each of the Lauge-Hansen groups (*p* > 0.05). Figure 4 shows an example of a “postero-lateral” type fracture which was stabilized with a cannulated screw using the described surgical technique, with a mean trajectory angle of 21°.

**Table 1:** Trajectory angles in each of the CT-based posterior malleolar fracture types

Fracture type	Number (%) of cases	Mean TA (range) (°)
Postero-medial	4 (4.7%)	28 (25–31)
Postero-central	20 (23.5%)	7 (2–13)
Postero-lateral	61 (71.8%)	21 (17–26)

For “postero-medial” fragments, the trajectory angle line is typically subtended from a starting point medial to the AP central line. For “postero-lateral” and “postero-central” fragments, the trajectory angle line is typically subtended from a starting point lateral to the AP central line

*TA* trajectory angle

**Table 2:** Trajectory angles in each of the Lauge-Hansen classification-based posterior malleolar fracture types

Fracture type	Number (%) of cases	Mean TA (range) (°)
Pronation abduction injury—type II	5 (5.9%)	18.4 (5.4–22.7)
Pronation abduction injury—type III	20 (23.5%)	19.4 (3.5–30.9)
Pronation lateral rotation injury—type IV	13 (15.3%)	20.7 (10.8–31.7)
Supination abduction injury—type II	1 (1.2%)	3
Supination lateral rotation injury—type III	8 (9.4%)	17.7 (2.1–22.9)
Supination lateral rotation injury—type IV	32 (37.6%)	18.1 (2.1–26.7)
Isolated posterior lip injury	6 (7.1%)	16.3 (4.4–22.3)

*TA* trajectory angle

## Discussion

The primary outcome of this study is a set of average trajectory angles for an ideal single percutaneous posterior-to-anterior lag screw fixation of posterior malleolar fractures, based on pre-operative axial CT images. The impetus for this study stems from the anecdotal and scientific evidence which demonstrates unsatisfactory functional outcomes in patients with ankle fractures including a posterior malleolar fragment [6, 14, 17]. In some cases, even limited posterior malleolar fragments are indicative of poor prognosis and portend an increased risk for the development of ankle arthritis [18, 19, 22]. While failure of treatment is commonly multi-factorial, in cases of posterior malleolar fractures, a substantial contributing factor to inferior outcomes and the progression of ankle arthritis may be the difficulty in obtaining anatomic close reduction of the posterior malleolar fragment. This may result in tibiotalar joint incongruity, which substantially increases joint contact pressure [13, 22, 23]. Moreover, mal-positioned screws in a posterior malleolar fracture could inadvertently cause mal-reduction of the syndesmosis and lead to separation of the fibula from the anatomical location in the incisura [10, 13, 16]. Fitzpatrick et al. [10] conducted a cadaveric study of 9 specimens with a supination-external rotation injury with a posterior malleolus fracture. The authors showed a direct link between syndesmotic mal-reduction and posterior malleolus mal-reduction. Screws that entirely miss the posterior malleolar fragment or do not capture the fragment well can lead to joint incongruencies or subluxation. As the extent and location of posterior malleolar fractures are optimally assessed on axial CT cuts and are commonly miss-appreciated on standard radiographs [12, 14]. We have presented how a preoperative CT scan can be used in order to avoid these hazardous surgical pitfalls.

As previously discussed in the introduction part, indications for surgery in cases of posterior malleolar fractures are inconclusive and usually rely on the size of the posterior malleolar fragment and the presence of posterior subluxation of the tibial-talar joint [1–6]. The extent and location of posterior malleolar fractures are optimally assessed on axial CT cuts and are commonly misappreciated on standard radiographs^12; 14; 24; 25^. Thus, reducing and fixing these fragments anatomically using only fluoroscopic-guided surgery becomes challenging. Nevertheless, it is not common for surgeons to use intra-operative CT in these cases [15]. Solan et al. [13] also advocated the use of a CT scan for a better understanding of the patterns of posterior malleolus fractures that extend medially and to ensure adequate reduction.

Previously, there have been attempts to categorize ankle fractures with a posterior fragment using CT imaging. Bartoníček et al. [24] divided the fractures into five categories, based on the size, shape, location of the fragment, stability of the tibiotalar joint, and the integrity of the fibular notch. A large number of types may be regarded as a disadvantage from a clinical perspective. In addition, the authors include in the analysis pilon fractures, which have a very different pathomechanism. Mason et al. [25] divided the fractures into 3 groups based on severity and pathomechanics. Type 1 is an extra-articular posterior malleolar fragment, type 2 involves a primary fragment of the Volkman area with or without a medial injury, and type 3 is a fracture in the coronal plane that includes all the plafond. The medial posterior fracture group in our series did not match any of these types.

Haraguchi et al. [14] were the first to divide and classified the posterior malleolus fracture based on a CT scan of 57 patients. The authors introduce 3 common patterns: posterolateral oblique which constituted 38% of all the types, medial extension (19%), and small-shell type (14%). The aim of the study was mainly descriptive to examine the ratio of the posterior fragment area to the total cross-sectional area of the tibial plafond, along with the angle between the bimalleolar axis and the major fracture line. In our study, we examined 85 CT scans, and based on Haraguchi et al., we further developed the classification and found 3 different fracture patterns with distinct mean trajectory angles which may be applicable in the operation theater using merely an intraoperative fluoroscopy. Furthermore, we compared the Lauge-Hansen classification system [20] to the posterior malleolus fracture pattern has seen on a CT scan and found it to be incompatible.

The Lauge-Hansen classification system for ankle fractures is commonly used in orthopedic trauma surgery and is based solely on plain radiographs. The classification was designed to describe the mechanism of injury; however, low accuracy demonstrated with this classification [7]. This study did not find a good correlation between the ideal trajectory angles and the Lauge-Hansen classification of each ankle fracture type. The mean trajectory angle by Lauge-Hansen classification was approximately 20°, demonstrating that the classification does not have a sensitivity to distinguish between different posterior malleolar fracture patterns. A preoperative or intraoperative CT scan can greatly assist in the anatomic articular reduction in these cases. Furthermore, the Lauge-Hansen classification does not differentiate nor describe the different patterns of the correlates with the area of the posterior malleoli fracture and thus does not correlate with the findings and the suggested modified classification of this study.

Utilizing axial CT cuts, we were able to categorize each posterior malleolar fracture into one of three major categories. The most common type was “postero-lateral,” which comprised about two thirds of the cases. This is consistent with a previous study, which showed that the most frequent type of posterior malleolus ankle fractures involved the postero-lateral part of the malleolus [14]. The mean trajectory angle required for properly intersecting and fixing this type of fragment was found to be approximately 21° (17–26°) in this series. Therefore, using a trajectory angle of 20° aiming laterally from the central antero-posterior line during surgery and using the antero-posterior and lateral fluoroscopic views as described may increase the odds of obtaining good fixation in the majority of “postero-lateral” fragments.

For “postero-medial” and “postero-central” types, the range of trajectory angles needed for a proper posterior malleolar fragment fixation was also relatively narrow. A trajectory angle of approximately 28° aiming medially from the central antero-posterior line may capture the majority of “postero-medial” fragments, and a trajectory angle of approximately 7° aiming laterally from the central antero-posterior line may capture the majority of “postero-central” fractures. As the mean trajectory angles for all three types are relatively reproducible, they may be a good intraoperative reference for surgeons to use in addition to fluoroscopic reduction and their primary ankle fracture fixation techniques. However, cautions should be used when applying this technique since in some situations, two posterior-to-anterior lag screws may be preferred, or a single screw in addition to a posterior buttress plate may be superior to a single lag screw. The mean trajectory angles that are provided in this paper are meant to guide a surgeon’s understanding of common fracture patterns and not to suggest that these fractures should ideally be fixed with a single anterior-to-posterior lag screw in any or all cases.

Although the presented categorization system could describe most posterior malleolus fractures in our series, four cases were comminuted and did not fit in any category. This is consistent with previous descriptions of posterior malleolar fractures [14] and implies that using the CT-based trajectory angle as described here may not be sufficient to provide anatomic reduction and mechanical stability of all fractures. In these cases, posterior buttress plating may be more appropriate than using screws alone. When the fragmented area is medial, the interval between the flexor hallucis longus and the Achilles tendon can be used for optimal surgical approach, and when the fragmented area is lateral, the interval between the peroneal tendons and the Achilles can be used. Moreover, combinations of “postero-lateral” and “postero-medial” fragments can occur in posterior malleolar fractures [14]. In these cases, it may be optimal to use at least two lag screws at the appropriate trajectory angles or posterior buttress plating.

### Study limitations

The limitations of this study include the lack of clinical outcome data to confirm the reproducibility of the surgical technique described. Furthermore, the cost and implications of increased radiation associated with CT imaging of all intraarticular ankle fractures are not insignificant. The different trajectory angles and their proximity to the surrounding neuro-vascular structures were not thoroughly examined in practice, and further investigation is needed. Nonetheless, we assumed a small skin incision followed by a minimal blunt dissection prior to the KW and screw insertion would provide sufficient protraction. Prospective controlled trials of different fixation methods and imaging techniques that include assessment of patient-reported clinical outcomes would further illuminate the effectiveness of different approaches to posterior malleolar fragment fixation.

#### Conclusion

This study defines three distinct and common anatomic subgroups of posterior malleolar fragments and defines the average trajectory angle for ideal posterior to anterior lag screw fixation of these subgroups. The use of CT imaging in intraarticular ankle fractures and specifically in posterior malleolar fractures can increase the understanding of this fracture pattern with the goal of improving anatomic fixation and ultimately, the clinical outcomes. Moreover, the Lauge-Hansen classification system may be inadequate to characterize specific variants of posterior malleolar fracture patterns and this should be taken into account during operative fixation of ankle fractures.

## Data Availability

Not applicable.

## Abbreviations

AP: Antero-posterior
CT: Computed tomography
rKW: Reference Kirschner Wire
tKW: Trajectory Kirschner Wire
